# Upregulated WTAP expression appears to both promote breast cancer growth and inhibit lymph node metastasis

**DOI:** 10.1038/s41598-022-05035-y

**Published:** 2022-01-19

**Authors:** Chao-Qun Wang, Chih-Hsin Tang, Yan Wang, Bi-Fei Huang, Gui-Nv Hu, Qian Wang, Jun-Kang Shao

**Affiliations:** 1 Department of Pathology, Affiliated Dongyang Hospital of Wenzhou Medical University, 60 Wu Ning Xi Road, Dongyang, 322100 Zhejiang People’s Republic of China; 2 Graduate Institute of Basic Medical Science, China Medical University, Taichung, Taiwan; 3 Department of Pharmacology, School of Medicine, China Medical University, Taichung, Taiwan; 4 Department of Biotechnology, College of Health Science, Asia University, Taichung, Taiwan; 5 Department of Medical Oncology, Affiliated Dongyang Hospital of Wenzhou Medical University, Dongyang, Zhejiang People’s Republic of China; 6 Department of Surgical Oncology, Affiliated Dongyang Hospital of Wenzhou Medical University, Dongyang, Zhejiang People’s Republic of China

**Keywords:** Biomarkers, Oncology

## Abstract

It is unclear as to whether Wilms’ tumor 1-associated protein (WTAP) promotes or suppresses breast cancer. This immunohistochemistry analysis explored levels of WTAP expression in 347 cases of breast cancer and analyzed the relationship between WTAP expression and the clinicopathological characteristics and prognosis of breast cancer patients. The rate of high WTAP expression was significantly higher in breast cancer tissue than in adjacent normal breast tissue (37.5% vs 0.0%; *P* < 0.001). WTAP expression was positively associated with tumor size and grade, and negatively associated with axillary lymph node metastasis, estrogen and progesterone receptor status. Rates of high WTAP expression were 66.1% in triple-negative breast cancer (TNBC) tissue and 31.3% in non-TNBC tissue. In multiple logistic regression analysis, independent predictors of WTAP expression in breast cancer included larger tumor size (odds ratio = 1.907; 95% confidence interval: 1.185–3.067; *P* = 0.008), lymph node metastasis (0.597; 0.373–0.956; *P* = 0.032) and TNBC status (3.735; 2.056–6.784; *P* < 0.001). No clear relationship was observed between patient prognosis and WTAP expression. We suggest that WTAP expression is upregulated in breast cancer and appears to both promote tumor growth and inhibit lymph node metastasis.

## Introduction

Female breast cancer is the most commonly diagnosed cancer worldwide and is the leading cause of cancer-related death among women^1,2^. Wilms’ tumor 1-associated protein (WTAP) is a key component of the methyltransferase complex, mediating in the deposition of cellular *N*^6^-methyladenosine (m^6^A), which is intimately involved in the initiation and progression of various types of human cancers^3,4^. In particular, WTAP contributes to aggressive features of malignant tumors. For example, WTAP overexpression facilitates the progression of hepatocellular carcinoma via the HuR-ETS proto-oncogene 1 (ETS1) axis^5^ and promotes osteosarcoma tumorigenesis by repressing HMBOX1 expression^6^. WTAP is also an oncogene in endometrial cancer^7^, gastric cancer^8^, ovarian cancer^9^ and bladder cancer^10^, which are all associated with poor prognosis.

Research on the expression of WTAP in breast cancer tissue and its clinical significance is only available from individual case reports, and contrasting findings have been reported from analyses of the expression and function of WTAP^11,12^. In an analysis of three human cancer databases^11^ that examined the relationship between WTAP expression and the prognosis of breast cancer patients, reduced WTAP expression was associated with poor survival in one database, and increased WTAP expression was associated with poor survival in another one database, while the results of the two microarrays in the third database are opposite, showing the same result of the first and second database above, respectively. In another analysis, WTAP expression was reduced in breast cancer samples compared with normal tissue derived from The Cancer Genome Atlas (TCGA) data^12^. Thus, further research is needed to better understand the expression and function of WTAP in breast cancer.

This study performed an immunohistochemistry (IHC) analysis of WTAP expression in breast cancer tissue samples obtained from 347 Chinese Han women, to clarify the expression of WTAP in breast cancer and its clinicopathological and prognostic significance.

## Results

### WTAP expression in breast tissue and its relationship with clinicopathological characteristics

**Figure 1:**
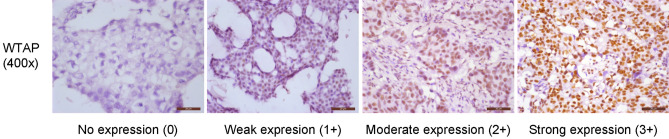
WTAP expression levels in breast cancer patients. Breast cancer tissue specimens were immune-stained with anti-WTAP antibody, photographed using an optical microscope and scored from 0 (negative) to 3 (strong) for nuclear staining intensity of WTAP expression.

WTAP was expressed in the nuclei of breast cancer cells. The proportion of high WTAP expression in breast cancer tissue specimens was 37.5% (130/347), compared with 0.0% (0/23) of normal breast tissue specimens (Fig. 1); the expression of WTAP in breast cancer was significantly higher than that in normal breast tissue (*P* < 0.001) (Table 1).

**Table 1:** WTAP expression in breast tissue specimens.

Tissue samples	No.	WTAP expression	
		Low expression, n (%)	High expression^†^, n (%)
Noncancerous	23	23 (100.0%)	0 (0.0%)
Cancerous	347	217 (62.5%)	130 (37.5%)*

^†^High WTAP expression was defined as a nuclear staining intensity of positive cancer cells of 3^13^. *P < 0.05 vs normal breast tissue.

As shown in Table 2, high WTAP expression was identified in 44.7% (84/188) of cases with tumors larger than 2 cm and in 28.9% (46/159) of tumors ≤ 2 cm; the between-group difference was highly significant (*P* = 0.003). High WTAP expression was identified in 54.9% (56/102) of grade III tumor tissue, in 30.7% (70/228) of grade II tumor tissue and in 23.5% (4/17) of grade I tumor tissue (*P* < 0.001). In patients with axillary lymph node metastasis and in those whose tumors were ER- or PR-positive, rates of high WTAP expression were 31.6% (54/171), 31.6% (68/215) and 29.6% (53/179), respectively, all of which were significantly lower than the corresponding rates among patients without lymph node metastasis (43.2%, 76/176), samples of ER-negative tissue (47.0%, 62/132) and PR-negative tissue (45.8%, 77/168) (*P* = 0.026, *P* = 0.004 and *P* = 0.002, respectively). Among the molecular subtypes that were examined, the rate of WTAP expression was 66.1% (41/62) in TNBC tissue compared with around one-second of non-TNBC tissue (31.3%, 88/281).

**Table 2:** Association of WTAP expression with clinicopathological parameters in breast cancer patients.

Variables	No. of patients	High WTAP expression, n (%)	*P*-value
**Age (years)**			
≤ 35	19	10 (52.6%)	0.373
36–55	202	74 (36.6%)	
> 55	126	46 (36.5%)	
**Tumor size (cm)**			
≤ 2	159	46 (28.9%)	0.003
> 2	188	84 (44.7%)	
**Lymph node metastases**			
No	176	76 (43.2%)	0.026
Yes	171	54 (31.6%)	
**Tumor grade**			
I	17	4 (23.5%)	< 0.001
II	228	70 (30.7%)	
III	102	56 (54.9%)	
**Tumor stage**			
I	94	28 (29.8%)	0.072
II–III	253	102 (40.3%)	
**Estrogen receptor**			
Negative	132	62 (47.0%)	0.004
Positive	215	68 (31.6%)	
**Progesterone receptor**			
Negative	168	77 (45.8%)	0.002
Positive	179	53 (29.6%)	
**HER2 expression**			
Negative (0–1^+^)	148	55 (37.2%)	0.982
Equivocal (2^+^)	110	42 (38.2%)	
Positive (3^+^)	89	33 (37.1%)	
**Molecular classification**			
Non-TNBC	281	88 (31.3%)	< 0.001
TNBC	62	41 (66.1%)	
**Lymphovascular invasion**			
No	118	61 (51.7%)	0.047
Yes	34	11 (32.4%)	

*High WTAP expression was defined as a nuclear staining intensity of positive cancer cells of 3^13^.

*HER2* human epidermal growth factor receptor 2, *TNBC* triple-negative breast cancer.

In logistic regression multivariate analysis, independent predictors of WTAP expression in breast cancer included larger tumor size (odds ratio = 1.907; 95% confidence interval: 1.185–3.067; *P* = 0.008), lymph node metastasis (0.597; 0.373–0.956; *P* = 0.032) and TNBC subtype (3.735; 2.056–6.784; *P* < 0.001).

We further analyze the relationship between WTAP expression and lymphatic invasion. The proportion of lymphovascular invasion (LVI) in breast cancer tissue specimens was 22.4% (34/152). High WTAP expression was identified in 32.4% (11/34) of LVI-positive tumor tissue and in 51.7% (61/118) of LVI-negative tumor tissue (*P* = 0.047). Spearman correlation analysis revealed a significantly negative correlation between WTAP expression and LVI in breast cancer tissue specimens (*r* = –0.161, *P* = 0.047).

### No association between WTAP expression and survival

**Figure 2:**
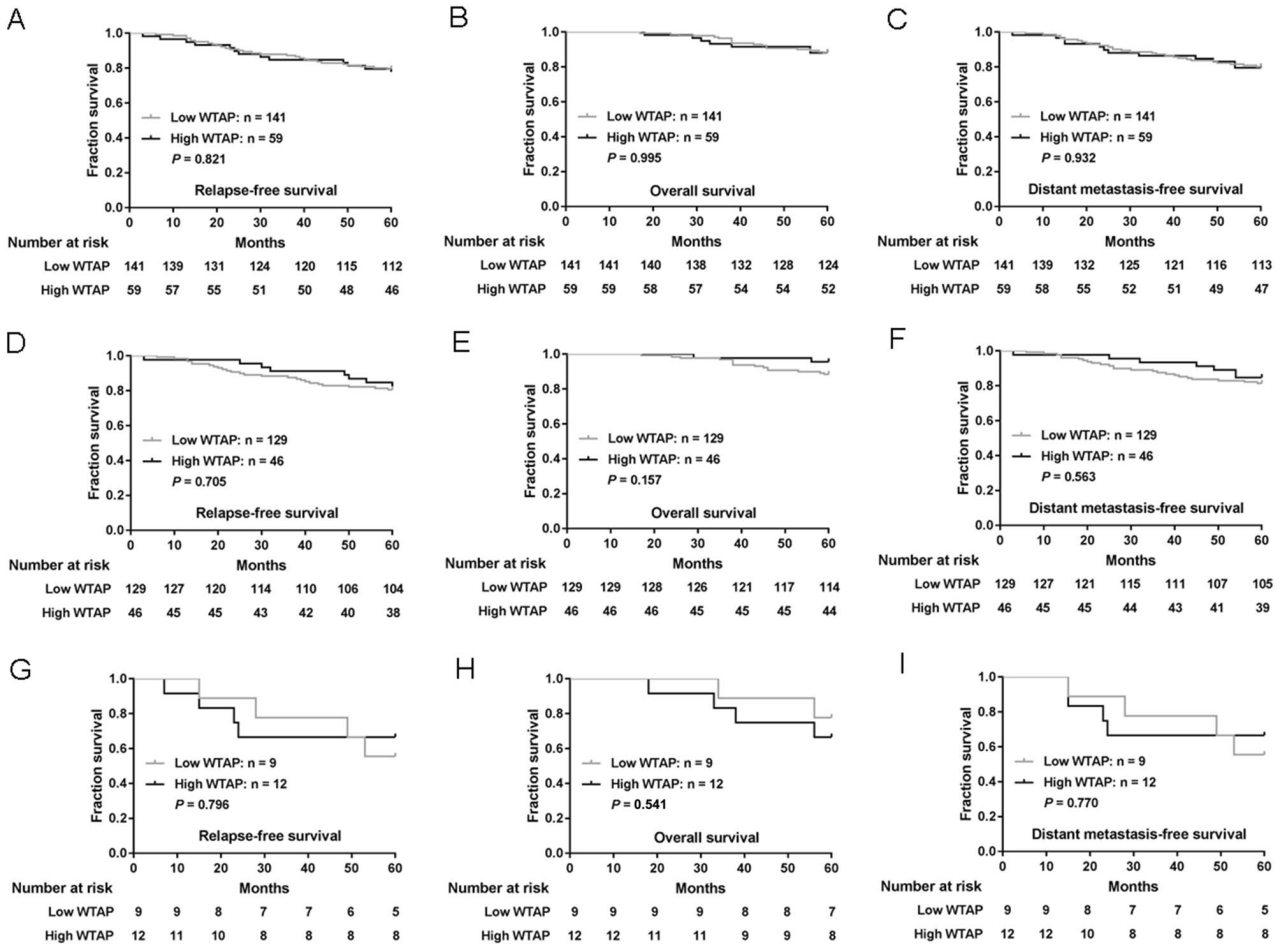
(**A**-**C**) The associations of WTAP expression with relapse-free survival (RFS) (A), overall survival (OS) (B) and distant metastasis-free survival (DMFS) (C). (**D**-**F**) The associations of WTAP expression with RFS (D), OS (E) and DMFS (**F**) of patients with non-TNBC. (**G**-**I**) The associations of WTAP expression with RFS (G), OS (H) and DMFS (I) of patients with TNBC. *P*-values were calculated using the Mantel-Cox log-rank test.

To assess the potential impact of WTAP expression on patient survival, we analyzed WTAP expression in relation to relapse-free survival (RFS), overall survival (OS) and distant metastasis-free survival (DMFS) rates. Five-year RFS, OS and DMFS rates were 79.0%, 88.0% and 80.0%, respectively. Local recurrence, regional recurrence, and distant metastasis occurred in 6 (3.0%), 2 (1.0%), and 34 (17.0%) patients, respectively. As shown in Fig. 2A–C, no clear associations were observed between WTAP expression and survival. The 59 patients with high levels of WTAP expression had a mean RFS of 53.3 months and an estimated 5-year RFS rate of 78.0%; corresponding values in the 141 patients whose tumors expressed low levels of WTAP were 53.8 months and 79.4%, respectively (*P* = 0.821; Fig. 2A). Mean OS was 57.3 months (with an estimated 5-year OS rate of 88.1%) in the patients with high levels of WTAP expression and 57.7 months (with an estimated 5-year OS rate of 87.9%) in those with low levels of WTAP (*P* = 0.995; Fig. 2B). Mean DMFS was 53.8 months (with an estimated 5-year OS rate of 79.7%) in the patients with high levels of WTAP expression and 54.1 months (with an estimated 5-year OS rate of 80.1%) in those with low levels of WTAP (*P* = 0.932; Fig. 2C).

We analyzed the effect of WTAP expression on the prognosis of non-TNBC or TNBC. As shown in Fig. 2D–F, in non-TNBC, the prognosis of tumors that were high WTAP expression did not differ significantly from that of low WTAP group. Similarly, in TNBC, the prognosis of tumors that were high WTAP expression did not differ significantly from that of low WTAP group (Fig. 2G–I).

## Discussion

Several studies have reported that WTAP plays a important role in promoting the occurrence and development of malignant tumors^5–10^. However, scant research is available regarding WTAP expression and function in breast cancer, with only two published reports^11,12^. Both examined human cancer databases to analyze the expression of WTAP in breast cancer and its effect on prognosis in breast cancer, but the results of these analyses are controversial^11,12^. Thus, the role and function of WTAP in breast cancer needs to be clarified with urgency.

Our findings revealed significantly upregulated WTAP expression in breast cancer tissue compared with noncancerous breast tissue, in larger- versus smaller-sized tumors, and in higher-grade tumors. Higher-grade breast cancers are known to be more aggressive and to be associated with a poor prognosis^14^. These findings indicate that WTAP may be closely related to the occurrence of breast cancer, to highly invasive breast cancer and a poor prognosis. Our results also revealed that WTAP expression was significantly upregulated in ER- and PR-negative disease. WTAP was closely and negatively correlated with hormone receptor expression. As hormone receptor-negative breast cancer patients are unsuitable for endocrine therapy, further research should examine whether these patients may benefit from WTAP knockdown. Interestingly, we also found that high WTAP expression was negatively associated with axillary lymph node metastasis. Further analysis showed that larger tumor size and TNBC subtype are independently positively correlated with WTAP expression, and that lymph node metastasis is independently negatively correlated with WTAP expression. Paltoglou et al.^15^ reported that transcription factor Grainyhead-like 2 (GRHL2) plays a multifaceted role in prostate cancer, enhancing the oncogenic androgen receptor (AR) signaling pathway and promoting tumor growth, as well as suppressing metastasis-related phenotypes by suppressing epithelial-mesenchymal transition and cell invasion. These results indicate that WTAP may exert a dual function of promoting breast cancer growth and inhibiting lymph node metastasis.

Previous studies have shown that WTAP overexpression facilitates the tumor growth and progression of hepatocellular carcinoma via the HuR-ETS1 axis^5^ and β-arrestin2 (ARRB2) promoted the growth and migration of colorectal cancer (CRC) cells by upregulating WTAP expression^16^. In addition, a study showed that WTAP promotes osteosarcoma tumorigenesis by repressing HMBOX1 expression in an m^6^A-dependent manner^6^. Whether WTAP may affect breast tumor growth through the above-mentioned molecular pathways needs further study. Our results showed that the expression of WTAP in TNBC is significantly higher than that in non-TNBC. Studies show that TNBC subtype had lower odds of LVI^17,18^ and axillary lymph node involvement^18–21^ relative to other subtypes. Therefore, we further analyzed the relationship between WTAP and LVI, and the result shows a negative correlation between high WTAP expression and LVI. It may be one of the explanations for the low incidence of lymph node metastasis in breast cancer with high WTAP expression.

Our survival analysis failed to reveal any association between WTAP expression and survival, which we suspect may be due to the oncogenic functions of WTAP being counterbalanced by its ability to suppress lymph node metastasis. Our study analyzed the relationship between WTAP and breast cancer growth and lymph node metastasis at the histological level, but have not explored the molecular mechanism of WTAP affecting breast cancer growth and lymph node metastasis. More research is needed to determine how WTAP exerts oncogenic functions and to clarify its molecular mechanisms that inhibit lymph node metastasis. Such knowledge is of important clinical significance for the future use of WTAP in the inhibition of breast cancer.

## Materials and methods

### Patients and tissue samples

Breast cancer tissue samples were obtained from 347 Chinese Han women who underwent breast cancer surgery in the Affiliated Dongyang Hospital of Wenzhou Medical University (Dongyang, Zhejiang, China) between 2007 and 2019. Contains 332 cases of invasive ductal carcinoma, 5 cases of mucinous carcinoma, 4 cases of medullary carcinoma, 4 cases of metaplastic carcinoma and 2 cases of invasive micropapillary carcinoma. Twenty-three samples of adjacent normal breast tissue were also obtained following surgical resection. Inclusion criteria: a patient’s surgical specimen was diagnosed with invasive breast cancer by pathological diagnosis; exclusion criteria: antitumor therapy such as targeted therapy, chemotherapy, immunotherapy and radiotherapy before surgery. Breast cancer patients were aged between 24 and 84 years, with a median age of 50 years. A pathohistological diagnosis was made according to breast tumor classification criteria of the World Health Organization^22,23^. Histological grading was based on the Scarff–Bloom–Richardson system^14^. According to estrogen receptor (ER), progesterone receptor (PR) and human epidermal growth factor receptor 2 (HER2) status, tissue samples were classified into triple-negative breast cancer (TNBC) (ER^−^, PR^−^, HER2^−^) or non-TNBC subtype^24–27^. Four cases with ER^−^, PR^−^, and HER2 2^+^ equivocal status did not undergo fluorescence in situ hybridization (FISH) were not classified into TNBC or non-TNBC groups. Follow-up information was available for 200 patients with a median follow-up time of 60 months (range 17–60 months). The study was approved by the Ethics Committee of the Affiliated Dongyang Hospital of Wenzhou Medical University (2020-YX-063). Written informed consent was obtained from all the participants. All of the study methodology satisfied the relevant guidelines and regulations issued by the Affiliated Dongyang Hospital of Wenzhou Medical University.

### Tissue array preparation and IHC analysis

Tissue Array Preparation: We followed the methods described by Wang et al.^28^. In brief, the Quick-Ray^®^ UT-06 (Unitma Co., Ltd., Seoul, Korea) tissue microarray system and the Quick-Ray premade recipient block (UB-06) wax model were used to prepare tissue specimens (1 mm in diameter). Two representative sites from each breast cancer tissue sample were selected for sampling. IHC Analysis: IHC staining of paraffin-embedded tissue sections used the Envision System (Dako, Glostrup, Denmark), as described previously^25,26^. Primary antibodies consisted of anti-WTAP rabbit monoclonal antibody (clone EPR18744, diluted at 1:3200; Abcam, Cambridge, England), ready-to-use anti-ER rabbit monoclonal antibody (clone SP1, Dako), ready-to-use anti-PR mouse monoclonal antibody (clone PgR636, Dako), ready-to-use anti-Podoplanin mouse monoclonal antibody (clone D2-40, Dako) and HercepTest (Dako). The secondary antibody was Dako’s HRP rabbit/mouse universal antibody (Dako, Glostrup, Denmark). The negative control was incubated with vehicle then with secondary antibody, without primary antibody.

### Assessment of staining

WTAP staining in breast tissues was practically uniform throughout all tumor cells, so we needed to only evaluate WTAP staining intensity. The intensity of nuclear staining for WTAP was assessed in breast tissue and scored on a 4-point scale from 0 (negative) to 1 (weak), 2 (moderate), or 3 (strong)^29,13^; high WTAP expression was defined as a nuclear staining intensity of 3^13^. A case was considered to be ER- or PR-positive when the percentage of positive invasive cancer cells (nuclear staining) was ≥ 1%^30^. HER2 status was determined by the 2018 American Society of Clinical Oncology/College of American Pathologists guidelines for HER2 testing in breast cancer^31^. Each entire section was scanned and scored independently by two pathologists.

### Patient follow-up

Patients were followed-up using previously described methods^27,28^. In brief, each patient was followed-up postoperatively by telephone call and thereafter at 6-monthly hospital appointments; follow-up was discontinued in the event of the patient’s death. A diagnosis of local breast cancer recurrence was made by clinical or histology results. Relapse-free survival (RFS) was defined as the time from surgery to relapse/metastasis; overall survival (OS) was the time from surgery to death (excluding non-tumor-related deaths); distant metastasis-free survival (DMFS) was the time from surgery to metastasis.

### Flow diagram

**Figure 3:**
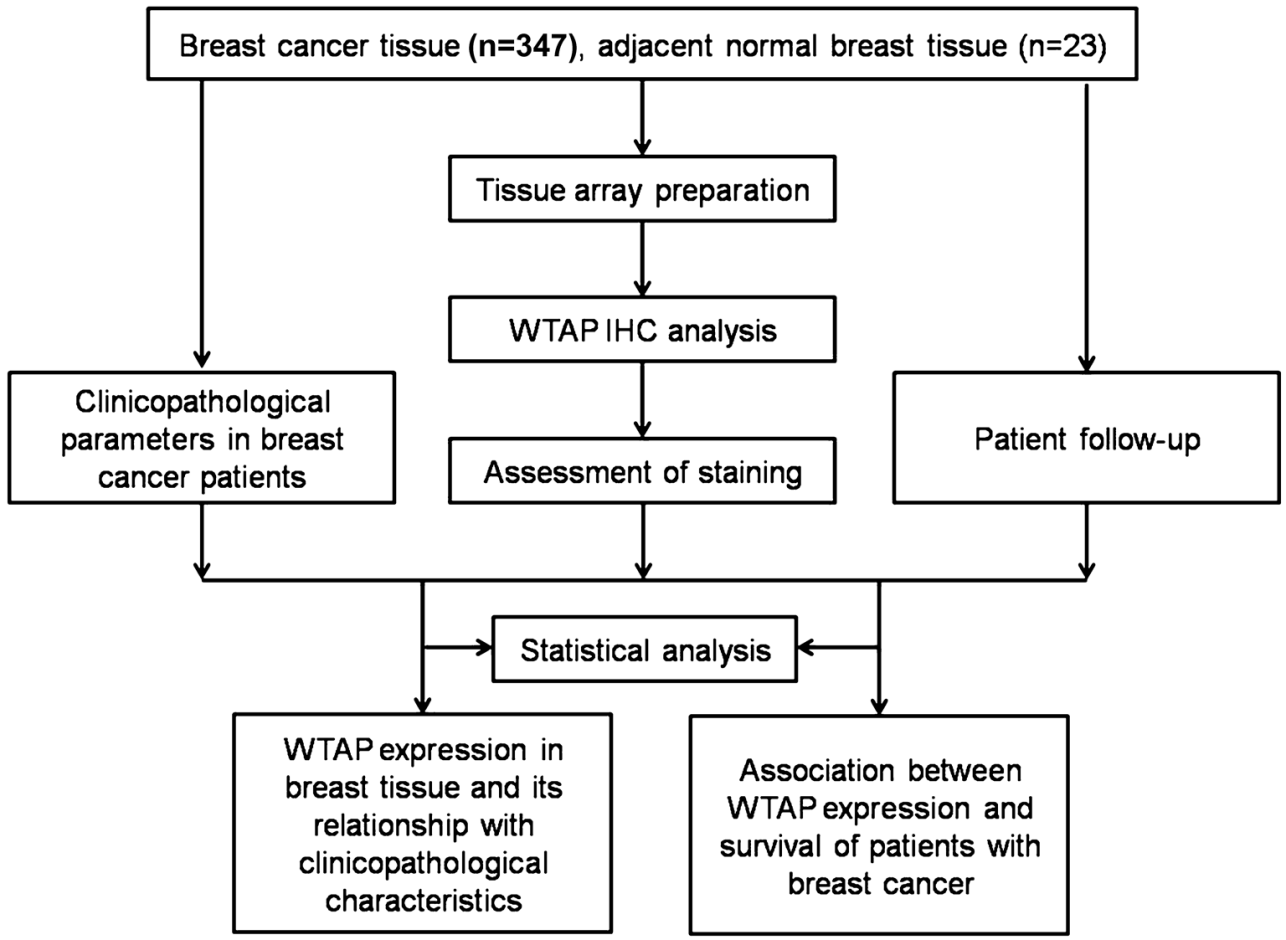
Flow diagram of the study methodology.

A flowchart of the study methodology is shown in Fig. 3.

### Statistical analysis

Statistical analyses were conducted using SPSS software version 19.0 (SPSS Inc, Chicago, IL, USA). Between-group differences of WTAP expression were compared using a Pearson’s chi-square test for qualitative variables. Independent correlation factors of WTAP expression were assessed by multivariate logistic regression analysis. Relapse-free survival (RFS) and overall survival (OS) rates were estimated by the Kaplan–Meier method and compared using log-rank testing. *P* < 0.05 was considered to be statistically significant.

## Data Availability

All data generated or analyzed during this study are included in this published article.

## Abbreviations

WTAP: Wilms tumor 1-associated protein
ER: Estrogen receptor
PR: Progesterone receptor
HER2: Human epidermal growth factor receptor 2
RFS: Relapse-free survival
OS: Overall survival
DMFS: Distant metastasis-free survival
IHC: Immunohistochemistry
TNBC: Triple-negative breast cancer
m^6^A: *N*^6^-Methyladenosine
